# Mitochondrial Retrograde Signaling: Triggers, Pathways, and Outcomes

**DOI:** 10.1155/2015/482582

**Published:** 2015-10-25

**Authors:** Fernanda Marques da Cunha, Nicole Quesada Torelli, Alicia J. Kowaltowski

**Affiliations:** ^1^Departamento de Bioquímica, Escola Paulista de Medicina, Universidade Federal de São Paulo, 04044-020 São Paulo, SP, Brazil; ^2^Departamento de Bioquímica, Instituto de Química, Universidade de São Paulo, 05508-000 São Paulo, SP, Brazil

## Abstract

Mitochondria are essential organelles for eukaryotic homeostasis. Although these organelles possess their own DNA, the vast majority (>99%) of mitochondrial proteins are encoded in the nucleus. This situation makes systems that allow the communication between mitochondria and the nucleus a requirement not only to coordinate mitochondrial protein synthesis during biogenesis but also to communicate eventual mitochondrial malfunctions, triggering compensatory responses in the nucleus. Mitochondria-to-nucleus retrograde signaling has been described in various organisms, albeit with differences in effector pathways, molecules, and outcomes, as discussed in this review.

## 1. Introduction

Mitochondria are believed to be former free-living bacteria that established a successful symbiosis with preeukaryotic cells billions of years ago [1]. Today, while being unquestionably essential for eukaryotic aerobic metabolism, they also exhibit multiple alternative functions, including the biosynthesis of intermediary metabolites, regulation of cytosolic Ca^2+^ homeostasis [2–6], and coordination of cell death [7–9], among others. Many age-induced processes (for review see [10]) and degenerative diseases (for review see [11]) are related to mitochondrial dysfunction, further highlighting the critical importance of this organelle.

The evolution of this endosymbiotic relationship between mitochondria and the host cell resulted in transfer of genetic material so that, currently, most mitochondrial proteins (but not all of them) are coded in the nucleus. In this scenario, the need for a communication system between mitochondria and the nucleus becomes evident, necessary not only to coordinate mitochondrial protein synthesis during biogenesis of the organelle, but also to communicate eventual mitochondrial malfunctions, triggering compensatory responses in the nucleus. This communication system was described to operate in various organisms and involves antegrade (nucleus to mitochondria), retrograde (mitochondria-to-nucleus) as well as intermitochondrial pathways [12]. Mitochondrial signaling continues to be studied and is uncovering a central role of mitochondria in an increasing number of homeostatic systems. This review focuses on retrograde signaling, discussing triggers, molecular pathways, and outcomes known so far. Special attention is devoted to mitochondrial-derived peptides as signaling molecules.

## 2. Mitochondrial Retrograde Signaling Pathways

**Figure 1:**
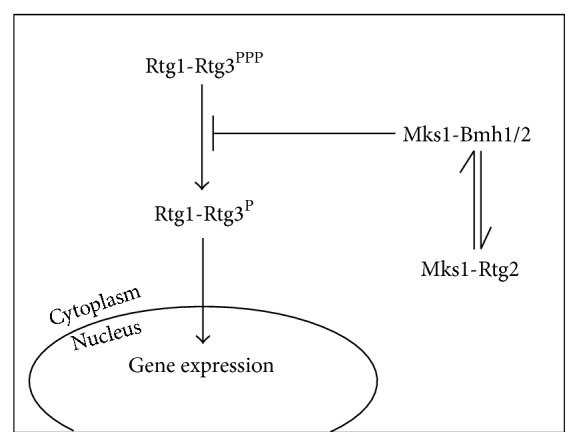
Simplified scheme of the RTG-dependent retrograde signaling pathway. In* Saccharomyces cerevisiae* this pathway depends on three proteins. Rtg1 and Rtg3 form a transcription factor that translocates to the nucleus when the pathway is activated. In the nucleus, Rtg1 and Rtg3 control the expression of a set of genes that code for mitochondrial proteins. Rtg2 is an activator of the pathway that allows the nuclear translocation of Rtg1 and Rtg3.

**Figure 2:**
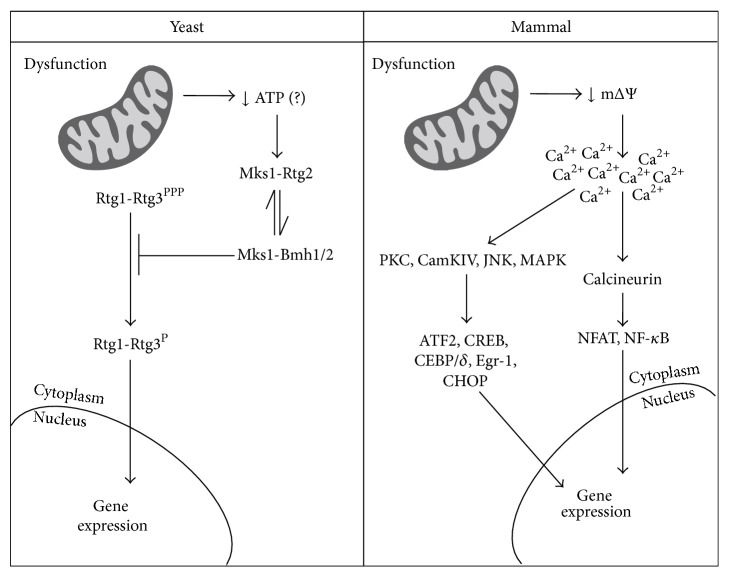
Scheme comparing the classical retrograde signaling pathways in yeast and mammals. In yeast, mitochondrial dysfunction leads to decreases in intracellular ATP concentration, which may favor Rtg2-Mks1 interaction [54] allowing Rtg1-Rtg3 activation. In mammals, mitochondrial dysfunction translates into drops in mitochondrial membrane potential, causing increments in intracellular calcium. Calcium-dependent kinases and phosphatases are then activated culminating with the activation of different transcription factors. Alternative retrograde signaling pathways in yeast, mammals, and other model organisms are discussed in the text.

*Saccharomyces cerevisiae*'s RTG-dependent retrograde signaling was the first retrograde pathway to be described and is extensively characterized [13, 14]. It depends on three cytosolic proteins: Rtg1p, Rtg2p, and Rtg3p. Rtg1p and Rtg3p are basic helix-loop-helix/leucine zipper (bHLH/LeuZip) transcription factors that bind as heterodimers to the GTCAC DNA binding site. When activated, the Rtg1/3p complex translocates from the cytoplasm to the nucleus [15], where it controls the expression of genes that encode mitochondrial proteins (Figures 1 and 2). Although only Rtg3p contains a transcription activation domain, Rtg1p and Rtg3p are both required for DNA binding [16].

Rtg1/3p translocation is dependent on partial dephosphorylation of Rtg3p [15]. Thus, inhibition of retrograde signaling occurs through the prevention of Rtg3p dephosphorylation mediated by Mks1p, a cytosolic phosphoprotein, when it is hyperphosphorylated and bound to Bmh1/2p (Figures 1 and 2). Rtg2p is an activator of the pathway that binds to the hypophosphorylated form of Mks1p, keeping it from binding to Bmh1/2p and allowing partial dephosphorylation of Rtg3p and Rtg1/3p translocation [17, 18]. Mks1p thus works through a dynamic switch between Rtg2p and Bmh1/2p: when bound to Rtg2p, retrograde signaling is active; when bound to Bmh1/2p, it is inactive. The Mks1p levels in the cell are controlled by SCF^Grr1^ E3 ubiquitin ligase-dependent polyubiquitination and degradation of free Msk1p, enhancing the efficiency of the Rtg2p/Bmh1/2p switch by keeping the concentration of free Mks1p low [19]. Rtg2p has an N-terminal HSP70-like ATP-binding domain that is required for the interaction with Mks1p [18]. In addition to its function as an activator of Rtg1/Rtg3p, Rtg2p is also a component of the transcriptional coactivator SAGA-like (SLIK) complex, which is required for* CIT2* expression, the prototypical reporter of RTG signaling [20].

In addition to coordinating the production of mitochondrial proteins, the retrograde signaling pathway has been found to coordinate carbon and nitrogen metabolism, since Rtg1/3p subcellular localization and activity are also regulated by the target of rapamycin (TOR) kinase pathway [21]. Inhibition of TOR function by rapamycin mimics nutrient starvation and affects genes involved in protein biosynthesis, the glycolytic pathway, the tricarboxylic acid cycle, and nitrogen metabolism, including permeases and degradation enzymes required for the use of different sources of assimilable nitrogen [22, 23]. Lst8p, a component of the target of rapamycin complex 1 (TORC1), is a negative regulator of the RTG-dependent retrograde signaling pathway [24] acting at two sites, one upstream of Rtg2p and one between Rtg2p and Rtg1/3p. Upstream regulation is believed to involve Lst8p in the activity or assembly of the SPS (Ssy1p, Ptr3p, and Ssy5p) amino acid-sensing system, affecting external glutamate sensing and consequently the retrograde response [25, 26]. The mechanism of Lst8p inhibition downstream of Rtg2p remains unknown. Treatment with rapamycin inhibits TOR function and thus activates retrograde signaling, inducing the expression of RTG-target genes [26]. It is also known that mitochondrial dysfunction leads to decreased phosphorylation and reduced activity of Sch9p [27], a target of TORC1 important for ribosome biosynthesis, cell-size control, inhibition of entry into the stationary phase, and translation initiation [28]. This is yet another possible link between retrograde signaling and the TOR kinase pathway.

Resistance to osmotic stress is also related to RTG-dependent retrograde signaling. Exposure to external hyperosmolarity activates Hog1 stress-activated protein kinase (SAPK), which controls several transcription factors such as Sko1p, Hot1p, Msn2p and Msn4p, and Smp1p. These in turn regulate the expression of stress-response genes. Expression of RTG-dependent genes is also induced under osmotic stress and is dependent on Hog1 SAPK. Hog1 SAPK binds to the Rtg1/3p transcription factor and allows its translocation to the nucleus. Although only the presence of Hog1 SAPK is required for Rtg1/3p nuclear translocation, its activity is necessary for the transcription factor to bind to the chromatin [29].

Despite the fact that it is more extensively described, RTG-dependent signaling is not the only pathway through which yeast mitochondria communicate with the nucleus. A number of genes whose transcription is altered in response to mitochondrial dysfunction are not under the control of Rtg proteins. Additionally, depending on the yeast strain or the culture condition, a different set of genes have their expression modified when compared to the genes affected by mtDNA depletion [30], the classical paradigm for RTG-dependent signaling activation. One example is the upregulation of the ATP-binding cassette protein Pdr5p, a multidrug resistance transporter, shown to be driven by the Pdr1p/Pdr3p transcription complex and not by the Rtg1p/Rtg3p complex [31].

Arnold et al. [32] reported another kind of retrograde signaling in null mutants for the i-AAA-protease coded by* YME1* when growing on respiratory substrates. Interestingly, the response was recapitulated by inhibition of the Fo-F1 ATP synthase in wild-type cells but was abrogated by respiratory chain inhibition as well as by membrane potential dissipation.

Mutant yeast strains with impaired mitochondrial proteostasis also seem to display active retrograde signaling independent of Rtg proteins. In a null mutant for a component of the large subunit of the mitochondrial ribosome (Afo1p), an alternative retrograde pathway was shown to be dependent on active TORC1 and the transcription factor Spf1p [33]. In mutants that lack Sov1p, a protein of the mitochondrial translation control module, the retrograde pathway was shown to require Sir2p and* PCN1* [34]. Mitochondrial proteostasis impairments were also shown to be central to the mitochondrial unfolded protein response (mtUPR), another kind of mitochondrial retrograde signaling pathway described in* Caenorhabditis elegans*,* Drosophila melanogaster*, and mammalian cells [35–37]. In* C. elegans*, perturbation of mitochondrial protein handling by either genetic or pharmacological means induced mitochondrial retrograde signaling culminating with the selective expression of mitochondrial matrix chaperones encoded in the nucleus [36]. Additional data indicate that mtUPR in* C. elegans *may also be activated by RNAi-mediated knockdown of factors required for mtDNA expression [37] as well as knockdown of some respiratory chain components [38–40]. The same is also true for* D. melanogaster*, for which experimental evidences are less numerous but nonetheless indicate the activation of mtUPR when different respiratory chain components are knocked out or down [41–43].

In mammalian cells, altered nuclear expression in response to mitochondrial dysfunction has long been reported [44, 45], with a number of signaling pathways implicated in this retrograde communication [46]. Indeed calcineurin, PKC, CamKIV, JNK, and MAPK (kinases) as well as the transcription factors ATF2, CEBP/*δ*, NFAT, CREB, Egr-1, CHOP, and NF*κ*B participate in mammalian mitochondrial retrograde signaling [46–49] (Figure 2). Interestingly, evidence of retrograde signaling was also reported at the level of whole mammalian organisms. Despite their dysfunctional mitochondria and increased reactive oxygen species (ROS) production, knockout mutants with reduced* MCLK1* activity (involved in coenzyme Q synthesis) or* SURF1* (COX assembly factor) have increased life span [50, 51] as well as significant resistance to brain damage following global cerebral ischemia-reperfusion injury [52] or excitotoxic insults [51]. Interestingly,* MCKL1* heterozygous mice also have enhanced immune function [53], suggesting a new outcome of mitochondrial retrograde signaling activation, further discussed below.

## 3. Triggers and Relay Molecules of Retrograde Signaling Pathways

Retrograde signaling must be triggered by a mitochondrial signal that in turn is relayed to one or more molecules that finally reach the nucleus. While the yeast RTG-dependent retrograde signaling is molecularly well characterized, the same is not true for other retrograde pathways. In this section, events that may function as triggers and relaying molecules will be discussed.

ATP is one of the main mitochondrial products and would be an obvious trigger molecule. Some evidences suggest that this may be the case, at least in specific situations. In yeast, Mks1p release from Rtg2p is dependent on ATP hydrolysis and is ATP-specific, suggesting that loss of mitochondrial DNA activates the pathway possibly through a decrease in ATP concentration, allowing Mks1p-Rtg2p association and Rtg1/3p nuclear translocation (Figure 2) [54]. While decreases in ATP concentrations may occur in drastic situations, retrograde signaling was shown to be active during normal replicative [55] or chronological [56] aging, situations in which decreases in ATP are less likely. Drops in mitochondrial membrane potential were shown to trigger the retrograde response during replicative aging [55], although the mechanism through which this decrease in potential is relayed to Rtg2p is not defined [57]. In mammalian cells, disruption of the mitochondrial membrane potential is also the main trigger of retrograde signaling, impairing mitochondrial Ca^2+^ uptake and causing an elevation in free Ca^2+^ in the cytoplasm [58–61]. This, in turn, activates Ca^2+^-dependent protein kinase C (PKC), CamKIV, JNK, and MAPK, which then activate the transcription factors ATF2, CEBP/*δ*, CREB, Egr-1, and CHOP [46]. Elevated Ca^2+^ levels also activate calcineurin, a calcium-dependent serine-threonine phosphatase that induces NFAT and NF-*κ*B, which is considered to have evolved from RTG-dependent retrograde signaling (Figure 2) [47, 48]. Importantly, the causal relationship between mitochondrial dysfunction and calcium signaling was established by studies in which the chelation of free calcium was sufficient to abolish downstream signaling [58, 61]. Although determinant for retrograde signaling activation in different organisms, overt alterations in mitochondrial membrane potential do not seem to be the trigger of RTG-dependent signaling in yeast grown in raffinose, since RTG signaling was shown to be active and confer acetic acid resistance with no detected changes in membrane potentials [62].

An interesting possibility raised by Arnold et al. [32] is that mitochondria-derived peptides are involved in the activation of retrograde signaling in yeast, under certain circumstances. They showed that deletion of* YME1*, coding for i-AAA-protease in the inner membrane, abolished peptide generation in the intermembrane space and led to biogenesis of the respiratory chain and the induction of nuclear genes with functions in mitochondrial gene expression. The mitochondrial membrane potential was shown to be essential for the response, since the induction of nuclear genes was abolished by antimycin (an inhibitor of electron transport chain) or CCCP (a mitochondrial uncoupler), suggesting that mitochondrial transport of a yet uncharacterized relay molecule is part of the process.

In* C. elegans* with reduced expression of* SPG7* (a mitochondrial protease), the mitochondrial unfolded protein response (mtUPR) was shown to be activated in a manner dependent on* HAF1*, a gene encoding a mitochondria-localized ATP-binding cassette transporter [63]. The mechanism involves the transport and degradation of the transcription factor Atfs1p, which is normally imported into mitochondria and degraded. During mitochondrial stress, the import efficiency is reduced, allowing the traffic of Atfs1p to the nucleus and the consequent alteration of transcription of components of mtUPR [64]. It would be interesting to check whether, in this case, alterations in mitochondrial membrane potential are the primary cause of transport impairment. It would also be interesting to know if the reported alterations in mitochondrial membrane potential in mammalian or yeast cells affect the import/export of proteins and peptides and if this would in turn affect downstream signaling pathways as described for* C. elegans* [63, 64].

In* D. melanogaster*, a mutant defective in coenzyme Q synthesis (*SBO* gene mutant) presents activation of mtUPR together with attenuation of the insulin/insulin-like growth factor signaling (IIS) pathway [42]. In a more recent study with mutants for muscle* NDUFS1*/*ND75*, a component of complex I, nonautonomous attenuation of insulin/insulin-like growth factor signaling was shown to be responsible for life span extension [43]. Interestingly, the increase in life span was suppressed by forced expression of catalase or glutathione peroxidase I, uncovering a pivotal role for H_2_O_2_ in the signaling pathway. ROS (the specific chemical species is not characterized) were also shown to be part of the mtUPR induced by knockdown of* CCO1* (a subunit of mitochondrial cytochrome oxidase) in* C. elegans*. Indeed, the lifespan extension of this mutant partially depends on mild increases in mitochondrial ROS, a production that in turn activated the hypoxia-inducible transcription factor Hif1p [65]. Neuronal-limited knockdown of* cco-1* activates the mtUPR in the intestine in a cell nonautonomous manner, influencing the whole organism [66]. Whether the relaying factor (coined mitokine) is some oxygen-derived species was not investigated, but, as shown for* D. melanogaster*, this may be a possibility. Indeed, mitochondrial ROS have been implicated in mitochondria-to-nucleus signaling in different organisms, regulating the expression of enzymes involved in oxidative detoxification ([67], for review see [56, 68–73]). Even though the pathway by which mitochondria-derived ROS induces a protective response was dubbed mitohormesis [74], it is nonetheless a form of mitochondrial retrograde signaling that involves a mitochondrially derived signal inducing alterations in nuclear gene expression.

Increasing evidence indicates that a number of short open reading frames (sORFs) in the mtDNA can give rise to biologically active peptides that differ from those mentioned above in that they are not the product of degradation of existent mitochondrial proteins. The most prominent example is humanin, a 21- or 24-amino-acid peptide (depending on whether the translation occurs in the mitochondrion or the cytoplasm, resp.), discovered in 2001 during a search for protective molecules against Alzheimer's disease [75]. It is interesting to note that expressed sequence tags related to humanin were also found in rat [76] and* C. elegans *[77], suggesting its biological relevance. Humanin and a G14S modified version (HNG) were shown to protect cells against most Alzheimer-relevant insults [77] without changing A*β* or fibril amounts [78]. Additionally, cultured cells died less after challenges with H_2_O_2_, CoCl_2_, or oxidized LDL when treated with humanin [79–81]. This peptide or its modified version (HNG) also proved to be active in murine models of cardiac isquemia/reperfusion injury and stroke, decreasing the infarcted area [82, 83].

In addition to its cytoprotective effects, humanin was also shown to affect metabolism. Indeed, Muzumdar et al. [84] demonstrated that centrally administered HNG sensitizes rats toward insulin signaling through STAT3 activation in the hypothalamus. The link is further supported by the finding that endothelial cells of ApoE-deficient mice on a hypercholesterolemic diet have increased humanin levels [85]. In the same line, Ames mice (that have decreased signaling through the GH/IGF-1 axis) have increased humanin levels while GH transgenic mice that have increased signaling through the GH/IGF-1 axis show the opposite effect [86]. Increased levels of humanin were detected in mouse myocytes after cardiac ischemia/reperfusion injury [83], the skeletal muscle of patients with MELAS (Mitochondrial Encephalomyopathy with Lactic Acidosis and Stroke-like Episodes) [87], and neurons and glia of the occipital lobe of patients with Alzheimer's disease [88]. While it is clear that stress conditions lead to increments in humanin levels, very little is known about humanin regulation.

Humanin is not the only metabolically active mitochondrial sORF-derived peptide. MOTS-c (mitochondrial open reading frame of the 12S rRNA-C), a 16-amino-acid peptide coded by sORF of the mitochondrial 12S rRNA, was recently found to regulate insulin sensitivity and metabolic homeostasis by acting primarily on skeletal muscle in mice. Importantly, treatment of mice with MOTS-c prevented not only age-dependent and high fat diet-induced insulin resistance but also diet-induced obesity. MOTS-c was detected in different mouse and rat tissues as well as in human and rodent circulation. Its levels were decreased after fasting in skeletal muscle, testes, and plasma, whereas brain and heart presented sustained levels [89].

Increasing evidences indicate that mitochondrial sORF-derived peptides are potent and evolutionarily conserved mitochondrial signals able to affect various physiological processes. Because of their relevance, further studies aiming to characterize the molecules and mechanisms involved in mitochondrial sORF-derived peptides expression and modulation are needed.

## 4. Outcomes of Retrograde Signaling Activation

Independently of the organism or the pathway activated, the hallmark of mitochondrial retrograde signaling is the modification of the expression of nuclear genes induced by a signal from mitochondria. In* S. cerevisiae* treated with oligomycin or in a null strain for the* YME1* gene, the activation of a retrograde pathway leads to the expression of a number of genes involved in mitochondrial biogenesis [32]. Similarly, RTG-dependent signaling was shown to alter the expression of several genes [30, 90], including* CIT1* (encoding mitochondrial citrate synthase),* CIT2* (peroxisomal citrate synthase),* PYC1 *(pyruvate carboxylase),* ACS1* (acetyl-coenzyme A synthetase),* ACO1* (aconitase),* IDH1/2* (NAD^+^-dependent isocitrate dehydrogenase), and* DLD3* (D-lactate dehydrogenase) [91–95], ensuring that there is sufficient glutamate for biosynthetic pathways in cells with reduced respiratory capacity. Indeed, cells with mutant alleles of* RTG1 *or* RTG2 *are unable to grow in acetate as the sole carbon source, a sign of a defective tricarboxylic acid cycle, and are auxotrophic for glutamate or aspartate. Obstructions in the tricarboxylic acid cycle alone do not lead to glutamate or aspartate auxotrophy since its precursors can be provided by intermediates of the glyoxylate cycle. However,* RTG1*,* RTG2*, or* RTG3 *deletion impairs both cycles, thus making cells unable to grow without glutamate or aspartate [92, 96]. Retrograde signaling was also shown to affect amino acid metabolism, since* RTG1* or* RTG3* deletion results in increased levels of polyamine biosynthetic intermediates (putrescine, ornithine, and spermidine). Polyamines are known to have cytoprotective effects against oxidative imbalance and thus may act as stress defense systems in cells lacking retrograde signaling, in which the levels of other stress-response metabolites such as glutathione and trehalose are reduced during the stationary phase [97]. Additional evidence shows that cells impaired in RTG-dependent signaling have decreased catalase and glutathione peroxidase activity in the stationary phase and are more vulnerable to oxidative insults due to decreased hormetic concentrations of H_2_O_2_ [56]. Thus, an optimal redox defense system seems to be an important outcome of RTG-dependent retrograde signaling activation.

Not surprisingly, activation of RTG-dependent retrograde signaling in yeast was reported to extend replicative lifespan [55, 98–100]. The mechanisms are not entirely clear but seem to involve* RAS2* [99] as well as a counteraction of negative effects that rise from the age-induced increase in the generation of extrachromosomal rDNA (ribosomal DNA) circles (ERCs) [55, 98, 100, 101].

Replicative lifespan increases are also a reported outcome of the activation of RTG-independent retrograde pathways. Indeed, the aforementioned null mutant for Afo1p showed increased replicative lifespan and resistance to oxidants, despite its inability to grow in respiratory media [33]. This is also the case for the null mutant for* SOV1*, in which the mutation impaired the growth on respiratory media but improved protein homeostasis, increased genomic silencing, and induced a Sir2p- and* PCN1*-dependent extension in replicative life span [34]. Interestingly, the life span extension of this* SOV1* mutant was demonstrated to be due to the absence of mitochondrial translation control module proteins rather than the loss of mtDNA or respiratory activity [34]. It would be interesting to check whether the response activated in* SOV1* mutants has some parallels with mtUPR described in* C. elegans* or* D. melanogaster*.

In* C. elegans*, the main reported outcome of mtUPR activation has been life span extension (for review see [102]). The causal relationship between mtUPR and longevity, however, was recently questioned [66, 103, 104], and more data are needed to clarify this point. The data available on* C. elegans* and mtUPR suggest that, in spite of having common features like, for example, the induction of Hsp6p and Hsp60p, the effectors and signaling pathways may display some specificity related to the nature and/or the location of mitochondrial disturbance, which in turn may alter the outcome of the response (i.e., induce or not a nonautonomous response).

Similarly to* C. elegans*, the main reported outcome of mtUPR activation in* D. melanogaster* is increased life span [41]. One interesting point, however, is that some long-lived mutant flies also presented attenuation of the insulin/insulin-like growth factor signaling pathway [42]. Recently this nonautonomous attenuation was shown to be responsible for life span extension of mutants for muscle* NDUFS1*/*ND75* (a component of complex I) with an attenuation in insulin/insulin-like growth factor signaling caused by increased expression of ImpL2p (that can bind and inhibit* Drosophila* insulin-like peptides) [43]. It would be interesting to verify whether the insulin/insulin-like growth factor signaling pathway also plays a role in lifespan extension in long-lived* C. elegans* mitochondrial mutants.

As mentioned earlier, mitochondrial proteolysis perturbation by Spg7p protease inhibition in* C. elegans* led to mtUPR that required the transcription factor Atfs1p [63, 64]. One of the outcomes of this phenomenon was the production of antimicrobial molecules such as the peptide Abf2p and the lysozyme Lys2p [64]. Pellegrino et al. [105] found that worms preexposed to* SPG7* RNAi to induce mtUPR were more resistant to the pathogen* P. aeruginosa* when compared to animals exposed to control RNAi. Improved immune performance was also observed in long-lived* MCKL1* heterozygous mice [53], as previously mentioned. Indeed, these mutants had better outcomes, including less hepatic damage, after* Salmonella typhimurium* and* Salmonella enteritidis* infection [53]. Inducers of such improved immune function were not determined, but it is tempting to speculate, based on findings with* C. elegans*, that antimicrobial molecules induced by mtUPR in* MCKL1* mutants may be involved.

## 5. Conclusions

**Figure 3:**
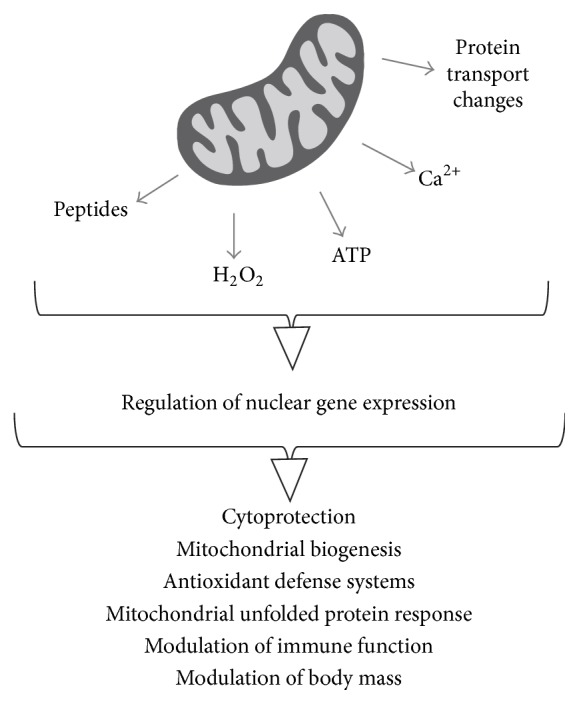
General view of mitochondrial signals and outcomes of retrograde communication. Diverse mitochondrial signals elicit varied responses, ranging from the increased synthesis of mitochondrial chaperones to improvement of immunity.

It has been a long time since mitochondria were believed to be organelles specifically responsible for ATP production. The vast array of data generated today indicate that mitochondria are metabolic hubs that detect and decode metabolic cues, generating signals which in turn are relayed by different molecules and pathways which finally reach the nucleus (Figure 3). Because of the diversity of mitochondria-derived signals, different retrograde communication pathways are employed to relay these signals to the nucleus.

It is interesting to note that the outcomes of mitochondrial retrograde signaling go far beyond the maintenance or biogenesis of the organelle, affecting the homeostasis of the whole organism through body weight or immunity, for example.
